# Supplementary material for: Epigenetic regulation of cocaine intake through dopaminergic control of cholinergic interneurons in male mice
Source: Nat Commun. 2025 Dec 9;16:10964. doi: 10.1038/s41467-025-65958-8 (PMC12689792; doi:10.1038/s41467-025-65958-8)
Supplement: Supplementary file 1 — Supplementary Information [file 41467_2025_65958_MOESM1_ESM.pdf]

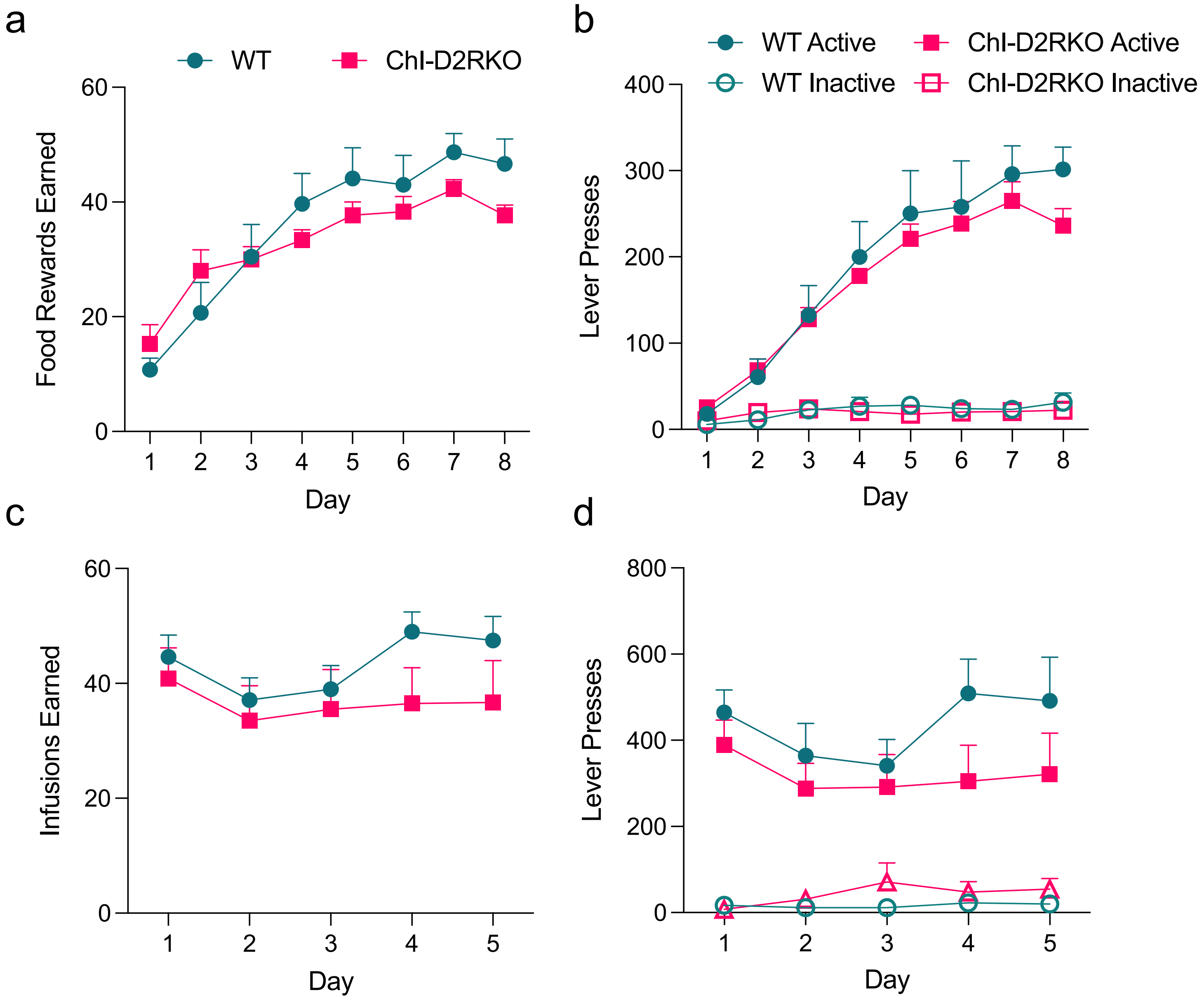

Supplemental Figure 1. ChI-D2RKO mice learn operant tasks as WT controls

(a) Number of food rewards earned and (b) lever presses during food training in WT (N=9) and ChI-D2RKO (N=7) mice during an FR5TO20 second schedule of reinforcement. (a) Two-way repeated measures ANOVA, session: $F_{(2.993,41.90)}$=22.15, P<0.0001; genotype: $F_{(1,14)}$=0.4823, P=0.4987; session × genotype: $F_{(7,98)}$=1.578, P=0.1510 and (b) Three-way repeated measures ANOVA, session: $F_{(3.164,88.59)}$=35.19, P<0.0001; genotype: $F_{(1,28)}$ =0.5062, P=0.4827; lever: $F_{(1,28)}$=108.0, P<0.0001; session × genotype: $F_{(7,196)}$=0.7318, P=0.6452; session × lever: $F_{(7,196)}$=28.54, P<0.0001; genotype × lever: $F_{(1,28)}$=0.3172, P=0.5778; session × genotype × lever: $F_{(7,196)}$=0.2900, P=0.9573. (c) Number of cocaine infusions earned and (d) lever presses after food training in WT (N=8) and ChI-D2RKO (N=6) mice during an FR5TO20 second schedule of reinforcement. (c) Two-way repeated measures ANOVA, session: $F_{(2.543,30.52)}$=3.165, P=0.0455; genotype: $F_{(1,12)}$=1.232, P=0.2888; session × genotype: $F_{(4,48)}$=1.275, P=0.2929 and (d) Three-way repeated measures ANOVA, session: $F_{(4,96)}$=2.610, P=0.0402; genotype: $F_{(1,24)}$=0.8339, P=0.3702; lever: $F_{(1,24)}$=50.45, P<0.0001; session × genotype: $F_{(4,96)}$=1.447, P=0.2245; session × lever: $F_{(4,96)}$=3.434, P=0.0114; genotype × lever: $F_{(1,24)}$=2.089, P=0.1613; session × genotype × lever: $F_{(4,96)}$=1.389, P=0.2434. Values shown are mean ± SEM.

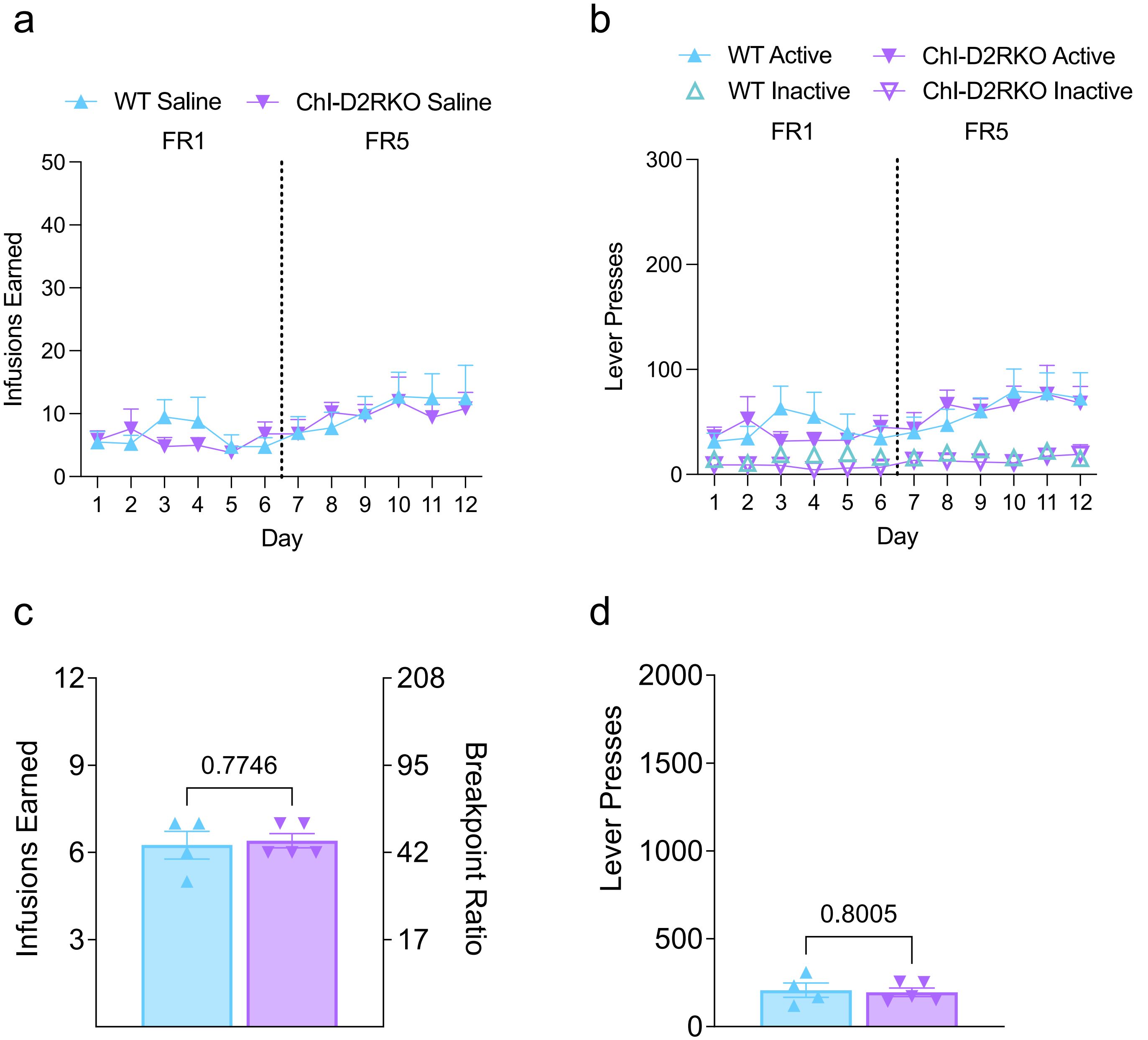

Supplemental Figure 2. Similar responses of ChI-D2RKO and WT mice to saline infusions
(a) Number of saline infusions earned and (b) lever presses during saline IVSA in WT (N=4) and ChI-D2RKO (N=5) mice during an FR1TO20 and FR5TO20 schedule of reinforcement. (a) Two-way repeated measures ANOVA, session: $F_{(2.585,18.10)}$=3.398, P=0.0458; genotype: $F_{(1,7)}$=0.09446, P=0.7675; session × genotype: $F_{(11,77)}$=0.6744, P=0.7583 and (b) Three-way repeated measures ANOVA, session: $F_{(1.883,13.18)}$=2.655, P=0.1096; genotype: $F_{(1,7)}$=0.2406, P= 0.6388; lever: $F_{(1,7)}$=24.54, P=0.0016; session × genotype: $F_{(11,77)}$=0.6934, P=0.7408; session × lever: $F_{(2.876,20.13)}$=2.305, P=0.1095; genotype × lever: $F_{(1,7)}$ =0.1017, p =0.7591; session × genotype × lever: $F_{(11,77)}$ =0.6888, P=0.7451. (b) Represents an average percent decrease compared to FR5 lever pressing for cocaine (Fig. 1b) of 189% for WT and 228% for ChI-D2RKO mice. (c) Number of saline infusions and (d) lever presses during a progressive ratio schedule of reinforcement in WT (N=4) and ChI-D2RKO (N=5). (c) Unpaired two-tailed t-test: P=0.7746, t=0.2977, df=7 and (d) Unpaired two-tailed t-test: P=0.8005, t=0.2624, df=7. Values shown are mean ± SEM.

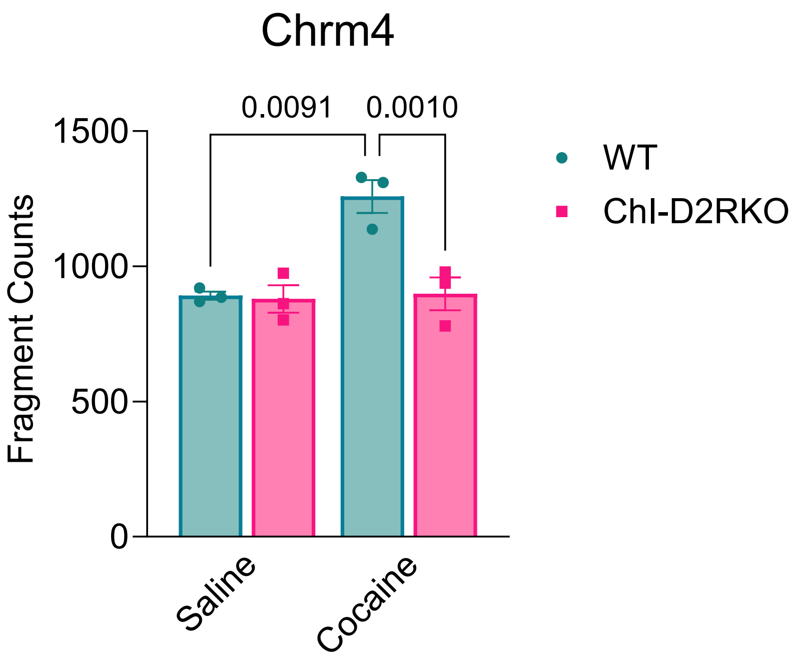

Supplemental Figure 3. Chrm4 expression in WT and ChI-D2RKO mice after PR
Normalized fragment counts of Chrm4 from DESeq2 (Fig. 2a,b) in the NAcc of WT and ChI-D2RKO mice (N=3/group) after the progressive ratio. Two-way ANOVA, genotype: $F_{(1,4)}=16.73$, $P=0.0150$; treatment: $F_{(1,4)}=12.37$, $P=0.0245$; genotype × treatment: $F_{(1,4)}=10.02$, $P=0.0340$. Values shown are mean ± SEM.

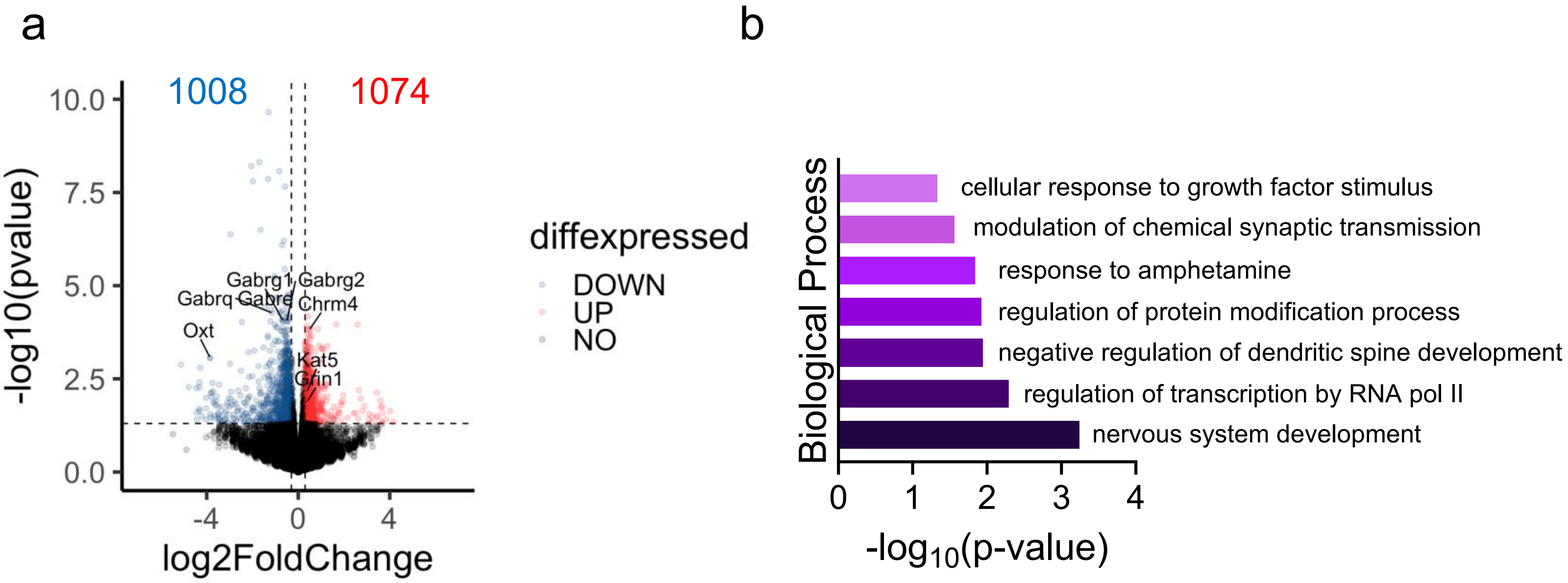

Supplemental Figure 4. Genotype-dependent cocaine responses in WT and ChI-D2RKO mice
(a) Volcano plot based on the -log10 p-value vs the Log2 Fold change of the cocaine responsive NAcc transcriptome (WT Cocaine vs ChI-D2RKO Cocaine; N=3/group). Cutoffs are shown as dotted lines ($P<0.05$) and log2FC ± 0.3. (b) PANTHER Gene Ontology based on differentially expressed genes from a.

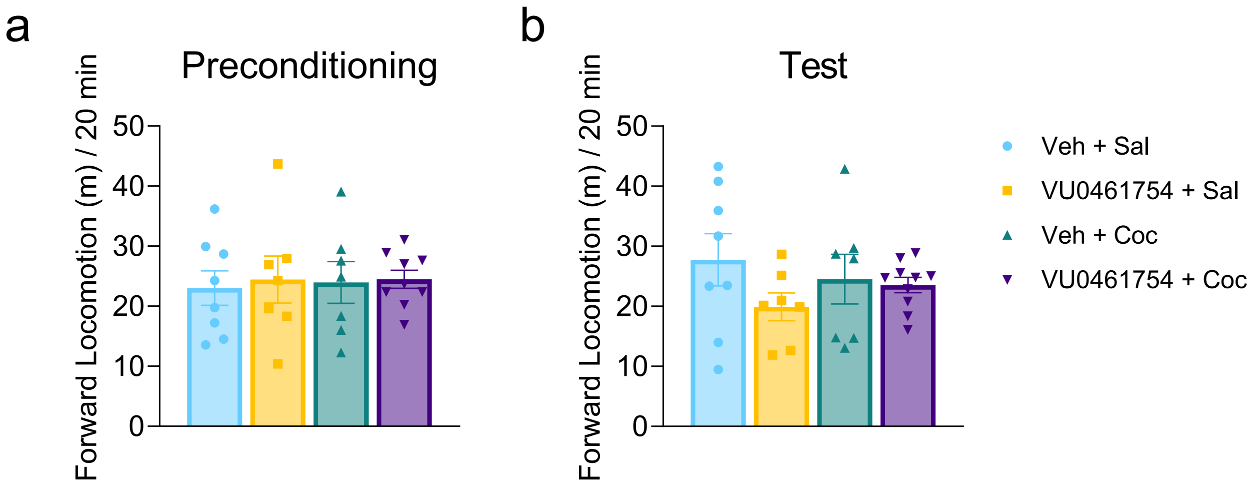

Supplemental Figure 5. Motor activity during preconditioning and test days of CPP
(a) Forward locomotion on the preconditioning day in WT mice assigned to be treated with vehicle or VU0467154 (5 mg/kg i.p.) prior to saline or cocaine during CPP conditioning (N=7-10/group). One-way ANOVA: $F_{(3,27)}$=0.05642, P=0.9820. (b) Forward locomotion on the CPP test day (N=7/10/group). One-way ANOVA: $F_{(3,28)}$=1.020, P=0.3985. Values shown are mean ± SEM.

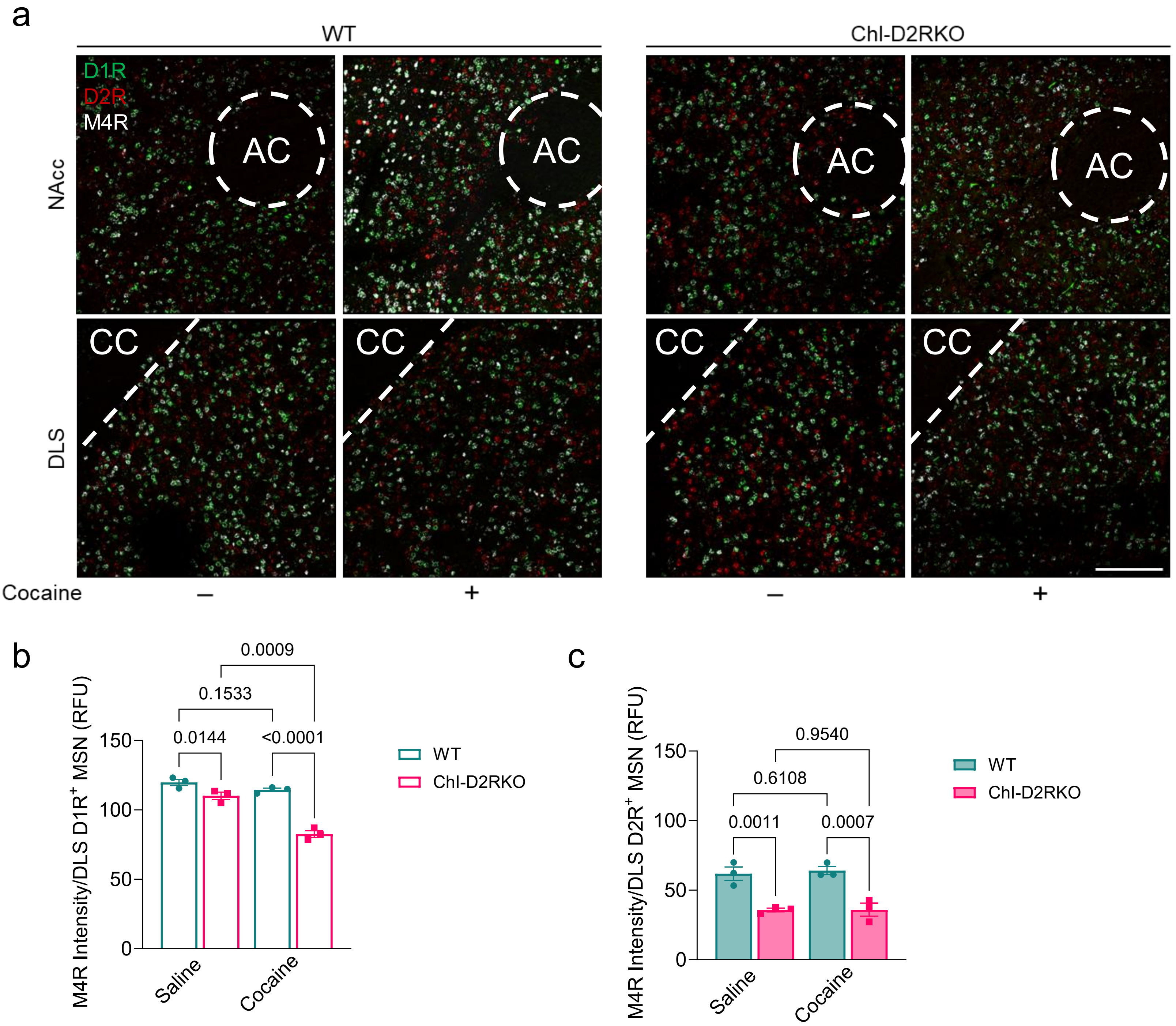

Supplemental Figure 6. Cocaine-induced upregulation of M4R in D1R+ MSNs is restricted to the ventral striatum

(a) Representative images of fluorescent in-situ hybridization (FISH) experiments showing D1R (green), D2R (red), and M4R (white) expression in the NAcc (top; Fig. 5a,c,e) and dorsal lateral striatum (DLS; bottom) of WT and Chl-D2RKO mice (N=3/group) treated with cocaine (15mg/kg i.p.) chronically for 7-days. Scale bar: 200μm. *Top:* the anterior commissure (AC) and *Bottom*: the lateral end of the corpus callosum (CC) are indicated. (b-c) Quantification of the fluorescent intensity (RFU) of M4R in (b) $D1R^+$ MSNs (Two-way ANOVA, genotype: $F_{(1,4)}=87.20$, $P=0.0007$; treatment: $F_{(1,4)}=57.61$, $P=0.0016$; genotype × treatment: $F_{(1,4)}=26.03$, $P=0.0070$) and (c) $D2R^+$ MSNs (Two-way ANOVA, genotype: $F_{(1,4)}=37.52$, $P=0.0036$; treatment: $F_{(1,4)}=0.1876$, $P=0.6872$; genotype × treatment: $F_{(1,4)}=0.1200$, $P=0.7465$) in the DLS. Values shown are mean ± SEM.

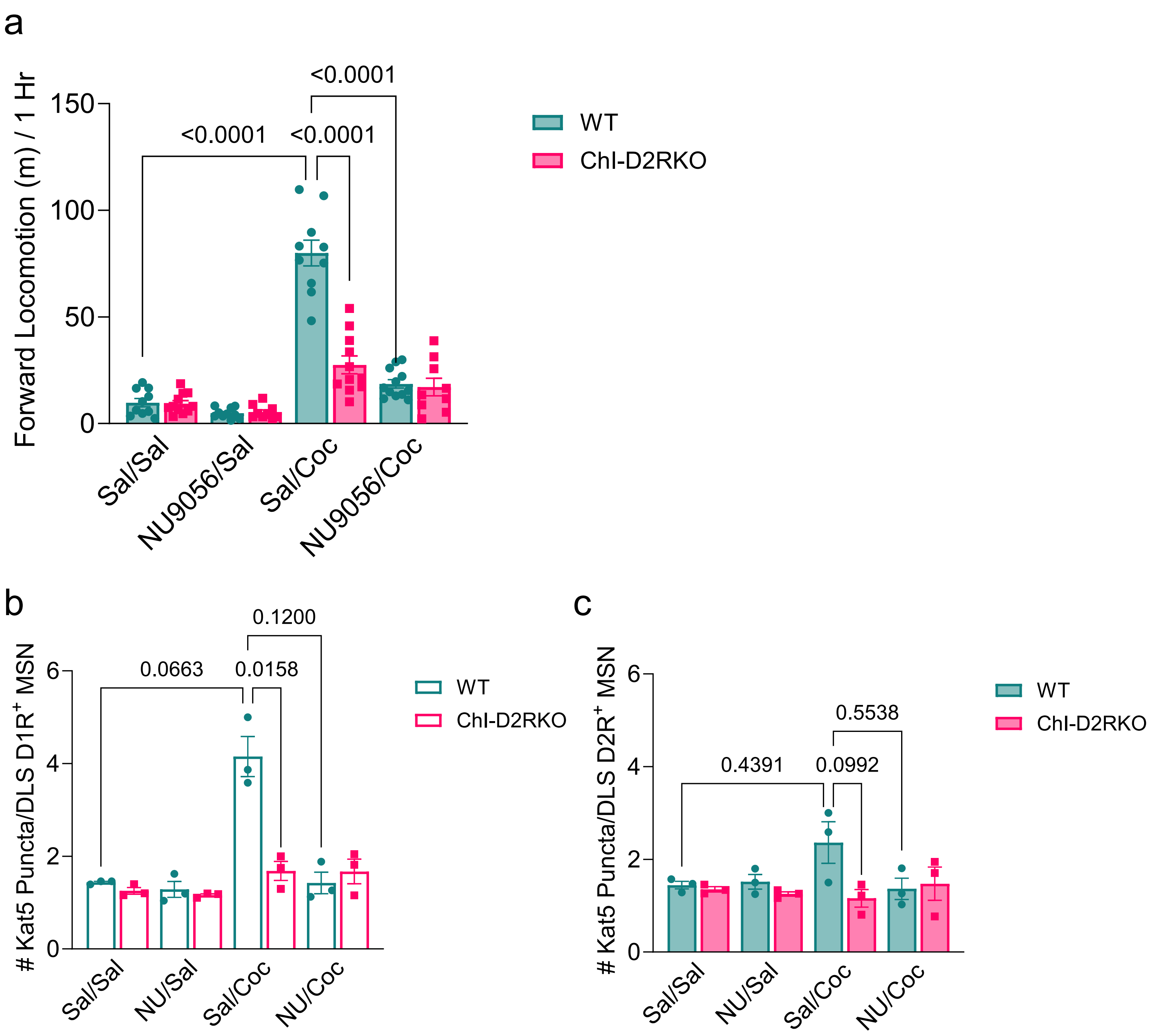

Supplemental Figure 7. Kat5 induction by cocaine in the $D1R^+$ MSNs of the DLS

(a) Forward locomotion in WT and ChI-D2RKO mice treated in acute with vehicle or NU9056 (2.5 mg/kg i.p.) 1-hour prior to saline or cocaine. Activity was recorded for 1-hour following saline or cocaine administration (N=9-12/group). Two-way ANOVA, genotype: $F_{(1,21)}=24.51$, $P<0.0001$; treatment: $F_{(1.746,30.48)}=116.4$, $P<0.0001$; genotype × treatment: $F_{(3,53)}=40.74$, $P<0.0001$. (b-c) Quantification of the number of $Kat5^+$ puncta in (b) $D1R^+$ MSNs (Two-way ANOVA, genotype: $F_{(1,4)}=18.55$, $P=0.0126$; treatment: $F_{(1.615,6.462)}=24.33$, $P=0.0013$; genotype × treatment: $F_{(3,12)}=15.13$, $P=0.0002$) and (c) $D2R^+$ MSNs (Two-way ANOVA, genotype: $F_{(1,4)}=5.151$, $P=0.0858$; treatment: $F_{(1.869,7.476)}=1.106$, $P=0.3749$; genotype × treatment: $F_{(3,12)}=2.857$, $P=0.0815$) in the DLS (N=3/group). Values shown are mean ± SEM.
