# Supplementary material for: Epigenetic regulation of cocaine intake through dopaminergic control of cholinergic interneurons in male mice
Source: Nat Commun. 2025 Dec 9;16:10964. doi: 10.1038/s41467-025-65958-8 (PMC12689792; doi:10.1038/s41467-025-65958-8)
Supplement: Supplementary file 2 — Reporting Summary [file 41467_2025_65958_MOESM2_ESM.pdf]

nature portfolio

Corresponding author(s): Emiliana Borrelli

Last updated by author(s): 10/6/2025

Reporting Summary

Nature Portfolio wishes to improve the reproducibility of the work that we publish. This form provides structure for consistency and transparency in reporting. For further information on Nature Portfolio policies, see our Editorial Policies and the Editorial Policy Checklist.

Please do not complete any field with "not applicable" or n/a. Refer to the help text for what text to use if an item is not relevant to your study. For final submission: please carefully check your responses for accuracy; you will not be able to make changes later.

Statistics

For all statistical analyses, confirm that the following items are present in the figure legend, table legend, main text, or Methods section.

n/a	Confirmed	
☐	☒	The exact sample size (*n*) for each experimental group/condition, given as a discrete number and unit of measurement
☐	☒	A statement on whether measurements were taken from distinct samples or whether the same sample was measured repeatedly
☐	☒	The statistical test(s) used AND whether they are one- or two-sided<br>*Only common tests should be described solely by name; describe more complex techniques in the Methods section.*
☐	☒	A description of all covariates tested
☐	☒	A description of any assumptions or corrections, such as tests of normality and adjustment for multiple comparisons
☐	☒	A full description of the statistical parameters including central tendency (e.g. means) or other basic estimates (e.g. regression coefficient) AND variation (e.g. standard deviation) or associated estimates of uncertainty (e.g. confidence intervals)
☐	☒	For null hypothesis testing, the test statistic (e.g. *F*, *t*, *r*) with confidence intervals, effect sizes, degrees of freedom and *P* value noted<br>*Give P values as exact values whenever suitable.*
☒	☐	For Bayesian analysis, information on the choice of priors and Markov chain Monte Carlo settings
☒	☐	For hierarchical and complex designs, identification of the appropriate level for tests and full reporting of outcomes
☒	☐	Estimates of effect sizes (e.g. Cohen's *d*, Pearson's *r*), indicating how they were calculated

Our web collection on statistics for biologists contains articles on many of the points above.

Software and code

Policy information about availability of computer code

Data collection	Mouse behavioral data was collected and analyzed using Lyon's ViewPoint software. Immunofluorescence images were captured using a Leica SP5 confocal, and western blot images were captured using a BioRad Chemidoc MP Imaging System.
Data analysis	Please see full list below.

For manuscripts utilizing custom algorithms or software that are central to the research but not yet described in published literature, software must be made available to editors and reviewers. We strongly encourage code deposition in a community repository (e.g. GitHub). See the Nature Portfolio guidelines for submitting code & software for further information.

Immunofluorescence images were analyzed using Imaris software (version 10.2.0) and western blot images were analyzed using Image Lab software (version 6.1). All statistical analyses were performed using GraphPad Prism software (version 10.2.3). RNAsequencing: read quality was assessed by FastQC, sequence alignment was performed using STAR 2.6.0c software, annotation and count matrices were generated using Genomic Features and Genomic Alignments, dDifferential expression analysis was performed using DESeq2, volcano plots were generated using the ggplot R package, and bological processes represented by the differentially expressed genes were determined using PANTHER Gene Ontology.

Data

Policy information about availability of data

All manuscripts must include a data availability statement. This statement should provide the following information, where applicable:

- Accession codes, unique identifiers, or web links for publicly available datasets
- A description of any restrictions on data availability
- For clinical datasets or third party data, please ensure that the statement adheres to our policy

All RNA-seq data is publicly available on ArrayExpress under acquisition code E-MTAB-14303 and in the NCBI Sequence Read Archive (SRA) under accession number PRJNA1338717. All other source data are provided with the article. Sharing of mice and other biological materials may be available upon request.

Research involving human participants, their data, or biological material

Policy information about studies with human participants or human data. See also policy information about sex, gender (identity/presentation), and sexual orientation and race, ethnicity and racism.

Reporting on sex and gender	N/A
Reporting on race, ethnicity, or other socially relevant groupings	N/A
Population characteristics	N/A
Recruitment	N/A
Ethics oversight	N/A

Note that full information on the approval of the study protocol must also be provided in the manuscript.

Field-specific reporting

Please select the one below that is the best fit for your research. If you are not sure, read the appropriate sections before making your selection.

☒ Life sciences ☐ Behavioural & social sciences ☐ Ecological, evolutionary & environmental sciences

For a reference copy of the document with all sections, see nature.com/documents/nr-reporting-summary-flat.pdf

Life sciences study design

All studies must disclose on these points even when the disclosure is negative.

Sample size	To estimate sample size we used the G*Power software and the PIs' prior studies with estimates for potential attrition and considerations to enhance reliability of the findings. Due to variability of behavioral experiments, we assume a moderate effect size f=0.25. For molecular experiments, we assume a large effect size f=0.40.
Data exclusions	For self-administration analyses, mice were wholly excluded due to death midway through the experimental timeline, or individual data points were excluded due to confounding behaviors such as escape maneuvers or chewing on tubing. No mice were excluded from any other data sets presented.
Replication	All experiments were successfully replicated using three or more independent biological replicates (animals) per experimental group and condition. For each experiment performed, the exact number of biological replicates used is specified in the figure legend.
Randomization	Sex and age-matched groups of male wild type and ChI-D2RKO littermates were used for all experiments presented. Mice were randomly distributed and assigned among experimental groups and conditions.
Blinding	Blinding was not possible for behavioral or self-administration experiments due to the need for operational controls across multiple research groups. Experimental groups and conditions were blinded as appropriate for molecular analyses.

Behavioural & social sciences study design

All studies must disclose on these points even when the disclosure is negative.

Study description	N/A
Research sample	N/A
Sampling strategy	N/A
Data collection	N/A
Timing	N/A
Data exclusions	N/A
Non-participation	N/A
Randomization	N/A

Ecological, evolutionary & environmental sciences study design

All studies must disclose on these points even when the disclosure is negative.

Study description	N/A
Research sample	N/A
Sampling strategy	N/A
Data collection	N/A
Timing and spatial scale	N/A
Data exclusions	N/A
Reproducibility	N/A
Randomization	N/A
Blinding	N/A

Did the study involve field work? ☐ Yes ☒ No

Field work, collection and transport

Field conditions	N/A
Location	N/A
Access & import/export	N/A
Disturbance	N/A

Reporting for specific materials, systems and methods

We require information from authors about some types of materials, experimental systems and methods used in many studies. Here, indicate whether each material, system or method listed is relevant to your study. If you are not sure if a list item applies to your research, read the appropriate section before selecting a response.

Materials & experimental systems

n/a	Involved in the study	
☐	☒	Antibodies
☒	☐	Eukaryotic cell lines
☒	☐	Palaeontology and archaeology
☐	☒	Animals and other organisms
☒	☐	Clinical data
☒	☐	Dual use research of concern
☒	☐	Plants

Methods

n/a	Involved in the study	
☒	☐	ChIP-seq
☒	☐	Flow cytometry
☒	☐	MRI-based neuroimaging

Antibodies

Antibodies used

Rabbit anti-Kat5: Protein Tech Cat# 10827-1-AP, 1:2000. Mouse anti-Tubulin: Protein Tech Cat# 66031-1-Ig, 1:10000. Mouse anti-Gapdh: Millipore Cat# MAB374, 1:10000. Rabbit anti-H4K8ac: Active Motif Cat# 61103, 1:300. Goat anti-rabbit HRP: Millipore Cat# AQ132P, 1:7500. Goat anti-mouse HRP: Millipore Cat# AP124P, 1:7500. AlexaFluor 647 goat anti-rabbit: Invitrogen Cat# A-21245, 1:750.

Validation

The references below provide additional information and validation with matching techniques and source materials for each of the primary antibodies used in this study.
Anti-Kat5: Pollina EA, Gilliam DT, Landau AT, Lin C, Pajarillo N, Davis CP, Harmin DA, Yap EL, Vogel IR, Griffith EC, Nagy MA, Ling E, Duffy EE, Sabatini BL, Weitz CJ, Greenberg ME. A NPAS4-NuA4 complex couples synaptic activity to DNA repair. Nature. 2023 Feb;614(7949):732-741. doi: 10.1038/s41586-023-05711-7. Epub 2023 Feb 15. PMID: 36792830; PMCID: PMC9946837.
Anti-Tubulin: Wang C, Xiong M, Gratuze M, Bao X, Shi Y, Andhey PS, Manis M, Schroeder C, Yin Z, Madore C, Butovsky O, Artyomov M, Ulrich JD, Holtzman DM. Selective removal of astrocytic APOE4 strongly protects against tau-mediated neurodegeneration and decreases synaptic phagocytosis by microglia. Neuron. 2021 May 19;109(10):1657-1674.e7. doi: 10.1016/j.neuron.2021.03.024. Epub 2021 Apr 7. PMID: 33831349; PMCID: PMC8141024.
Anti-Gapdh: Min SW, Chen X, Tracy TE, Li Y, Zhou Y, Wang C, Shirakawa K, Minami SS, Defensor E, Mok SA, Sohn PD, Schilling B, Cong X, Ellerby L, Gibson BW, Johnson J, Krogan N, Shamloo M, Gestwicki J, Masliah E, Verdin E, Gan L. Critical role of acetylation in tau-mediated neurodegeneration and cognitive deficits. Nat Med. 2015 Oct;21(10):1154-62. doi: 10.1038/nm.3951. Epub 2015 Sep 21. PMID: 26390242; PMCID: PMC4598295.
Anti-H4K8ac: Dansu DK, Sauma S, Huang D, Li M, Moyon S, Casaccia P. The epigenetic landscape of oligodendrocyte progenitors changes with time. bioRxiv [Preprint]. 2024 Feb 6:2024.02.06.579145. doi: 10.1101/2024.02.06.579145. PMID: 38501119; PMCID: PMC10946295.

Eukaryotic cell lines

Policy information about cell lines and Sex and Gender in Research

Cell line source(s)	N/A
Authentication	N/A
Mycoplasma contamination	N/A
Commonly misidentified lines (See ICLAC register)	N/A

Palaeontology and Archaeology

Specimen provenance	N/A
Specimen deposition	N/A
Dating methods	

☐ Tick this box to confirm that the raw and calibrated dates are available in the paper or in Supplementary Information.

Ethics oversight	N/A

Note that full information on the approval of the study protocol must also be provided in the manuscript.

Animals and other research organisms

Policy information about studies involving animals; ARRIVE guidelines recommended for reporting animal research, and Sex and Gender in Research

Laboratory animals	ChI-D2RKO mice were generated by mating D2Rfloxflox mice (used as WT controls) with choline acetyltransferase (ChAT)-Cre mice generating D2Rfloxflox/ChAT Cre/+ mice. All mice are bred in the Borrelli lab on a C57BL/6J background and were used at 8-16 weeks of age.
Wild animals	No wild animals were used for these studies.
Reporting on sex	Only male mice were used for all experiments. Sex as a biological variable (SABV) was not considered for the present study.
Field-collected samples	These studies did not involve field-collected samples.
Ethics oversight	All protocols were submitted and approved by the University of California, Irvine Institutional Animal Care and Use Committee in accordance with the National Institute of Health guidelines.

Note that full information on the approval of the study protocol must also be provided in the manuscript.

Clinical data

Policy information about clinical studies
All manuscripts should comply with the ICMJE guidelines for publication of clinical research and a completed CONSORT checklist must be included with all submissions.

Clinical trial registration	N/A
Study protocol	N/A
Data collection	N/A
Outcomes	N/A

Dual use research of concern

Policy information about dual use research of concern

Hazards

Could the accidental, deliberate or reckless misuse of agents or technologies generated in the work, or the application of information presented in the manuscript, pose a threat to:

No	Yes	
☒	☐	Public health
☒	☐	National security
☒	☐	Crops and/or livestock
☒	☐	Ecosystems
☒	☐	Any other significant area

Experiments of concern

Does the work involve any of these experiments of concern:

No	Yes	
☒	☐	Demonstrate how to render a vaccine ineffective
☒	☐	Confer resistance to therapeutically useful antibiotics or antiviral agents
☒	☐	Enhance the virulence of a pathogen or render a nonpathogen virulent
☒	☐	Increase transmissibility of a pathogen
☒	☐	Alter the host range of a pathogen
☒	☐	Enable evasion of diagnostic/detection modalities
☒	☐	Enable the weaponization of a biological agent or toxin
☒	☐	Any other potentially harmful combination of experiments and agents

Plants

Seed stocks

Novel plant genotypes

Authentication

ChIP-seq

Data deposition

☐ Confirm that both raw and final processed data have been deposited in a public database such as GEO.

☐ Confirm that you have deposited or provided access to graph files (e.g. BED files) for the called peaks.

Data access links
May remain private before publication.
N/A

Files in database submission
N/A

Genome browser session
(e.g. UCSC)
N/A

Methodology

Replicates
N/A

Sequencing depth
N/A

Antibodies
N/A

Peak calling parameters
N/A

Data quality
N/A

Software N/A

Flow Cytometry

Plots

Confirm that:

- [ ] The axis labels state the marker and fluorochrome used (e.g. CD4-FITC).
- [ ] The axis scales are clearly visible. Include numbers along axes only for bottom left plot of group (a 'group' is an analysis of identical markers).
- [ ] All plots are contour plots with outliers or pseudocolor plots.
- [ ] A numerical value for number of cells or percentage (with statistics) is provided.

Methodology

Sample preparation	N/A
Instrument	N/A
Software	N/A
Cell population abundance	N/A
Gating strategy	N/A

- [ ] Tick this box to confirm that a figure exemplifying the gating strategy is provided in the Supplementary Information.

Magnetic resonance imaging

Experimental design

Design type	N/A
Design specifications	N/A
Behavioral performance measures	N/A

Imaging type(s)	N/A
Field strength	N/A
Sequence & imaging parameters	N/A
Area of acquisition	N/A

Diffusion MRI ☐ Used ☐ Not used

Preprocessing

Preprocessing software	N/A
Normalization	N/A
Normalization template	N/A
Noise and artifact removal	N/A
Volume censoring	N/A

Statistical modeling & inference

Model type and settings	N/A
Effect(s) tested	N/A

Specify type of analysis: ☐ Whole brain ☐ ROI-based ☐ Both

Statistic type for inference (See Eklund et al. 2016)	N/A
Correction	N/A

Models & analysis

n/a	Involved in the study
☒	☐ Functional and/or effective connectivity
☒	☐ Graph analysis
☒	☐ Multivariate modeling or predictive analysis

Functional and/or effective connectivity	N/A
Graph analysis	N/A
Multivariate modeling and predictive analysis	N/A

This checklist template is licensed under a Creative Commons Attribution 4.0 International License, which permits use, sharing, adaptation, distribution and reproduction in any medium or format, as long as you give appropriate credit to the original author(s) and the source, provide a link to the Creative Commons license, and indicate if changes were made. The images or other third party material in this article are included in the article's Creative Commons license, unless indicated otherwise in a credit line to the material. If material is not included in the article's Creative Commons license and your intended use is not permitted by statutory regulation or exceeds the permitted use, you will need to obtain permission directly from the copyright holder. To view a copy of this license, visit http://creativecommons.org/licenses/by/4.0/
